# Exogenous preculture with sucrose and abscisic acid improves post-cryopreservation survival of eastern bracken fern gametophytes

**DOI:** 10.1038/s41598-023-45941-3

**Published:** 2023-10-28

**Authors:** Bo-Kook Jang, Sewon Oh, Daeil Kim, Ju-Sung Cho, Cheol Hee Lee

**Affiliations:** 1 Department of Horticulture, Sunchon National University, Suncheon, 57922 Republic of Korea; 2 Fruit Research Division, National Institute of Horticultural and Herbal Science, Rural Development Administration, Wanju, 55365 Republic of Korea; 3 Division of Animal, Horticultural and Food Sciences, Chungbuk National University, Cheongju, 28644 Republic of Korea; 4 Brain Korea 21 Center for Bio-Health Industry, Chungbuk National University, Cheongju, 28644 Republic of Korea

**Keywords:** Plant biotechnology, Plant development, Plant reproduction

## Abstract

Cryopreservation is an important technique used in the conservation of various plant tissues. This study proposes a cryopreservation method for the long-term conservation of eastern bracken fern gametophytes (*Pteridium aquilinum* var. *latiusculum*). Encapsulation–dehydration of the gametophytes was performed, and the exogenous sucrose and abscisic acid (ABA) preculture conditions were investigated. Gametophytes are sensitive to dehydration and drying, and the following treatment conditions were applied: encapsulation by alginate containing 0.75 M sucrose, 18-h loading treatment with 0.75 M sucrose, and 6-h drying treatment. The survival rate following cryopreservation was determined. The water content of < 27.5% in the alginate beads after dehydration and drying was found to be appropriate for ensuring survival. Additionally, performing an exogenous sucrose and ABA preculture was essential before encapsulation to achieve a survival of ≥ 90%. The high stress induced by cryopreservation and exogenous preculture regulated the expression of *PaSuSy*, *PaLEA14*, and *PaABI1b* and the endogenous ABA content. In eastern bracken gametophytes, *ABI1* appears to be a negative regulator of ABA signaling. These results indicate that the encapsulation–dehydration method is effective for the long-term conservation of eastern bracken fern gametophytes, and exogenous preculture alleviates abiotic stress and increases the survival rate.

## Introduction

Gene banks favor cryopreservation over other techniques as it enables the safe preservation of various plant tissues (such as seeds, spores, shoot tips, somatic embryos, and calluses) in a small space. Although well-established cryopreservation protocols exist for the safe preservation of many vegetatively propagated crops^1^ such as potatoes^2^, apples, pears^3^, and ornamental horticulture plants^4,5^, it can also be applied for the preservation of a wide range of flora including ferns^6–10^.

An important criterion for successful cryopreservation is the prevention of intracellular ice crystal formation. This is achieved using plant vitrification solutions (PVS). Various PVSs such as PVS2^11^ and PVS3^12^ have been developed, and they primarily comprise sucrose, glycerol, and ethylene glycol in a liquid basal medium. These components improve freezing- and desiccation tolerance, which improves survival after cryopreservation. Additionally, various cryopreservation methods such as encapsulation–dehydration^13^, cryo-plates^14^ and cryo-mesh^15^ have been developed to improve the survival ratio^16^. The encapsulation–dehydration method is based on a new vitrification-based protocol that was developed in the 1990s^13,17^. Briefly, samples are precultured in media containing high sucrose concentrations, gelled with an insoluble calcium alginate layer, and dehydrated prior to cryopreservation. This technique improves freezing- and desiccation tolerance and significantly lowers the water content in the samples^18^. Particularly, sucrose and abscisic acid (ABA) are primarily used for fern preculture. The effect of using 10 μM of ABA during fern preculture has been investigated for several fern species such as *Davallia fejeensis*, *Drynaria quercifolia*, *Cibotium glaucum*, *Adiantum trapeziforme*, *Adiantum tenerum*, *Polypodium aureum*^19^, and *Selaginella uncinata*^20^. A preculture medium containing 0.25 M sucrose and 10 μM ABA was found to enhance the survival rate of *Asplenium cuneifolium* Viv^21^ and *Cyathea australis*^22^ following cryopreservation.

Desiccation and freezing during cryopreservation can adversely affect tissue survival and regrowth. Plants counteract freezing-induced damage and protect the cells by accumulating sugars in response to low temperatures^23^. However, high levels of exogenous sugars can significantly increase the freezing tolerance of cells^24^. ABA is a phytohormone that controls tolerance to drying and protects plant cells from dehydration^25^. Furthermore, sucrose and ABA regulate the expression of key genes associated with freezing and desiccation stresses^26–29^. Sucrose synthase (SuSy) and sucrose phosphate synthase (SPS) are enzymes that play key roles in plant sugar metabolic pathways^30^. SuSy converts sucrose to glucose or fructose, which increases soluble carbohydrate accumulation, possibly resulting in improved stress tolerance^31^. Moreover, the expression of sucrose-related genes such as *SuSy* and *SPS* change in response to abiotic stresses (e.g. salt, drought, and cold stress)^32,33^. Abscisic acid insensitive (*ABI*) is a key transcription factor in the ABA signaling pathway that is directly involved in dehydration tolerance^34^. ABA-induced protein kinase (serine/threonine-protein kinase) and *ABI1* also play major roles in the ABA signaling pathway^35,36^. Furthermore, plant seeds inherently possess a strong drying tolerance, which enables them to withstand late maturation stages that are characterized by the rapid decrease in water content owing to the expression of late embryogenesis abundant (LEA) proteins^37^. These proteins exert osmoprotectant effects or desiccation tolerance^38^. They have been studied in moss and ferns^24^. For example, Khandelwal et al.^39^ reported that freezing and desiccation tolerance is enhanced by exogenous ABA in *Physcomitrella patens* protonemata. Additionally, gene expression levels of *LEA* and *ABI* increased under these conditions.

Other previous studies primarily focused on enhancing the regeneration and survival of fern gametophytes by enhancing preculture conditions. However, this study goes further by examining the relationship between the expression of genes involved in freezing and desiccation tolerance and endogenous ABA levels while using sucrose and ABA during preculture. We believe that these results would fill the knowledge gap in the research on storage physiology for the better long-term conservation of ferns. Therefore, we propose an efficient cryopreservation method for eastern bracken (*Pteridium aquilinum* var. *latiusculum*) gametophytes. We also tried to determine the conditions that would enhance survival through the use of exogenous sucrose and ABA preculture. Additionally, this study explores the accumulation of endogenous ABA in gametophytes as a direct result of the preculture conditions and reports on the expression of the related genes.

## Methods

### Plant materials

Sporophylls of *P. aquilinum* var. *latiusculum* (eastern bracken) were collected in a greenhouse at Chungbuk National University, Cheongju, Korea, in September 2018 (36°37′29.1″N, 127°27′17.1″E). No approval or permission was required for the collection of these samples (sporophylls). Spore disinfection and germination were performed according to the methods described by Jang et al.^40^. Briefly, a spore solution (1 mg·mL^−1^) was centrifuged (3 min, 1811×*g*), and the supernatant was removed. The spore was sterilized with 1.4% (v/v) sodium hypochlorite (Yuhanclorox Co., Ltd., Hwaseong, Korea) for 13 min and washed thrice with sterilized water. Thereafter, the spores were inoculated in Knop medium and germinated at 25 °C under a 16/8-h photoperiod with a light intensity of 30 μmol·m^−2^·s^−1^. Subsequently, the gametophytes obtained from the spores were subcultured on double-strength MS basal medium at 8-week intervals. These were used for further experiment.

### Encapsulation, loading, and drying

Encapsulation–dehydration methods were used to assess the effect of the encapsulation treatment on survival during cryopreservation. The encapsulation–dehydration process was performed according to the procedures described by Fabre and Dereuddre^13^ and Kulus et al.^41^ with necessary minor modification. Unencapsulated (untreated) and encapsulated samples were prepared, and their survival ratio was compared after the loading and drying processes. The survival of the gametophytes was analyzed with respect to the loading (0, 12, 18, and 24 h) and drying (0, 2, 4, and 6 h) time durations. For the encapsulation–dehydration process, 1 g of gametophytes was mechanically fragmented using a scalpel and mixed with 40 mL of calcium-free liquid MS medium containing 0.75 M (255 g·L^−1^) sucrose and 30 g·L^−1^ sodium alginate (CAS 9005-38-3; Duksan Company, Ansan, Korea). Then, the mixture was taken in a 100-mL plastic syringe (Zhejiang Huafu Medical Equipment Co., Ltd., Jiaxing, China); encapsulated gametophyte beads were obtained by dripping the mixture as droplets into 100 mM sterilized CaCl_2_ solution (CAS 10035-04-8; Daejung Chemicals & Metals Co., Ltd., Siheung, Korea). Next, the alginate beads were immersed in an Erlenmeyer flask containing 100 mL of sterilized loading solution (calcium-free liquid MS medium with 0.75 M sucrose) and cultured with shaking (125 rpm). Later, the alginate beads were removed from the loading solution, placed on two sheets of filter paper, and dried in a laminar flow on a clean bench (wind velocity, 0.3–0.45 m·s^−1^). The dried alginate beads were placed into a 2-mL cryotube and exposed to liquid nitrogen (LN) for 1 h. The LN-exposed cryotubes were thawed at 25 °C for 30 min and the gametophytes were subsequently cultured for 4 weeks in MS medium. Finally, the recovered gametophytes were cultured at 25 °C under a fluorescent lamp (30 μmol·m^−2^·s^−1^) and a 16/8-h photoperiod. The method described here was used to perform all encapsulation–dehydration experiments in this study.

### Determination of water content in encapsulated gametophytes at various loading and drying time durations

The water content of the encapsulated beads was measured before exposure to LN. Measurements were performed for 15 alginate beads per treatment in three replicates (*n* = 45). The weight of the alginate beads containing fragmented gametophytes was determined first. Next, the weight of the alginate beads that were dried for 72 h in a hot air dryer (60 °C) was calculated. These two values were used to determine the water content of the encapsulated gametophytes.

### Preculture with sucrose and ABA

Preculture was performed to enhance survival during cryopreservation. Gametophytes were subcultured in 2MS medium containing sucrose (0, 0.4, 0.7 and 1.0 M) or ABA (10 or 100 μM) and precultured for 72 h. These gametophytes were evaluated for survival under the previously described encapsulation–dehydration conditions (with encapsulation, 18 h loading, and 6 h drying). All survival ratios were evaluated by observing 10 encapsulated gametophytes after cryopreservation, and five replicates of the examination were performed. Furthermore, the surviving gametophytes were subjected to 4 weeks of recovery culture and then subcultured in 2MS growth medium for 4 weeks. The increase in fresh weight was determined for the gametophytes. The normal proliferation of gametophytes was evaluated by measuring the weight per alginate bead, which included the weight of the surviving gametophytes. The gametophyte weight determination in the samples was performed for ten replicates. The weight of fresh alginate beads, which includes the weight of fragmented gametophytes before treatment, was 74 ± 7 mg.

### RNA extraction, reverse transcription PCR and *PaSuSy*, *PaABI1b*, and *PaLEA14* gene expression analysis

Non-treated fresh gametophytes (control) and precultured samples were immersed immediately in LN. The collected samples were finely ground using a mortar and pestle with LN. At least 3 g of gametophytes were used for each extraction. RNA extraction was performed as per the protocols reported by Chang et al.^42^ and Gasic et al.^43^ Assessment of RNA integrity and concentration was performed using a DS-11 spectrophotometer (DeNovix Inc., Wilmington, DE, USA). cDNA was synthesized from the extracted RNA samples using the QuantiTect Reverse Transcription Kit (Qiagen, Hilden, Germany) by following the manufacturer’s protocols. The target genes (*PaSuSy*, *PaSPS*, *PaABIb1*, and *PaLEA14*) were selected from the Blast2GO data provided by Der et al.^44^ The primer sequences for performing RT-qPCR were designed using Primer3 version 0.4.0 (https://bioinfo.ut.ee/primer3-0.4.0/) (Table 1). Actin (*PaAct1*, F_5′-AGCTCTTGCTCGAAGTCCAA-3′, R_5′-CCACATGCTATCCTCCGTCT-3′, product size 168 bp, Tm 60 °C) was used as the reference gene. Reverse transcription PCR was performed using 300 ng/uL of cDNA and primer pairs for the four target genes and reference gene. PCR amplicons were confirmed using 1.5% agarose gel electrophoresis for 1 h. The agarose gel images were cropped, and uncropped images are provided in Supplementary Information. Real-time quantitative PCR (RT-qPCR) was performed as described by Oh et al.^45^. The relative gene expression for each treatment sample was calculated based on to the expression level of the control. The relative gene expression of each sample was examined in three replicates.

**Table 1:** Primer pairs for target genes used in this study.

Name^z^	Primer sequence (5′-3′)	Product size (bp)	Annealing temperature (℃)
*PaSuSy*	F-CTCGCTTCAGCACCATTGTA	190	62
	R-ACAGGTACATTGCGGACACA		
*PaSPS*	F-GGTTGATGAAAACCCCCTTT	226	62
	R-AATGTCGGCCATTGAGAGAG		
*PaABIb1*	F-CGAACATGGACAAGGAGGTT	177	60
	R-CCCCACAATTTGCAACTACC		
*PaLEA14*	F-TGAGCCTTGTCTTCGTCCTT	157	62
	R-TCTAGGGCTTCTCTCCGTCA		

^z^Refers to the Blast2GO data of Der et al.^44^.

### Endogenous ABA content in precultured encapsulated gametophytes

ABA analysis was performed by following the protocols described by Pan et al.^46^. The gametophytes that were used to analyze the endogenous ABA content were obtained after 24 h of recovery culture and washing with distilled water. To extract the gametophyte samples from each treatment, 50 mg of the ground sample that was obtained after freeze-drying was placed in a 2-mL tube. To this, 500 μL of the extraction solvent [2-propanol/H_2_O/concentrated HCl (2:1:0.002, v/v/v, %)] was added. Then, the sample was homogenized at 100 rpm and 4 °C for 30 min. To each homogenized sample, 1 mL of dichloromethane was added and shaken further for 30 min (100 rpm, 4 °C) and centrifuged for 5 min (13,000×*g*, 4 °C) (Smart R17 Plus; Hanil Scientific Inc., Gimpo, Korea). After the separation of the layers, 900 μL of the supernatant was transferred into a new 2-mL tube. The supernatant was evaporated using vacuum concentrators, and the recovered sample was dissolved in 100 μL methanol for further use. The standard solution used was 2-cis,4-trans-abscisic acid, which was dissolved in 1 mL methanol and diluted to a concentration of 1 mg·mL^−1^.

High-performance liquid chromatography (HPLC) was performed using an Agilent 1260 series system (Agilent Technologies, Palo Alto, CA, USA). Chromatographic separation was performed on an Agilent Eclipse Plus C_18_ column (4.6 × 50 mm, 3.5 μm particle size; Agilent Technologies, Palo Alto, CA, USA). The HPLC mobile phase was 0.1% formic acid in water (A) and 0.1% formic acid in methanol (B). The gradient was first started at 5% (B), increased to 95% (B) for 1 min, and maintained at 95% (B) for 4 min before being rapidly changed to 5% (B) for 0.1 min and maintained for 0.9 min. The flow rate, column oven temperature, and injection volume were 500 μL·min^−1^, 30 °C, and 10 μL, respectively.

An API-4000 (SCIEX, Framingham, MA, USA) mass spectrometer instrument equipped with an electrospray ionization source was used in the negative and MRM modes. BioAnalyst version 1.6.1 and Analyst software version 1.6.1 were used for equipment operation and data analysis, respectively. During ionization, high-purity nitrogen gas was used as the spray and drying gas at a gas pressure of 60 psi. The ion spray voltage and an ionization source temperature were − 4.5 kV and 600 °C, respectively. Q1 and Q3 were analyzed using LC–MS/MS multiple reaction monitoring using unit resolution. Analysis of the standard solution and each sample was performed in triplicate.

### Data collection and statistical analysis

Gametophyte survival was observed using a microscope (SZ61; Olympus Corporation, Tokyo, Japan). Gametophyte images were captured using a CMOS camera (eXcope F630; Dixi Sci., Daejeon, Korea) and eXcope 3.7.12277 software. Data are presented as the mean ± standard error for each treatment. Factorial analysis was performed using Duncan’s multiple range test using SAS version 9.4 (SAS Institute Inc., Cary, NC, USA), and significance was set at *P* < 0.05. Survival data (encapsulation, loading time, and drying time) and water content (loading and drying times) were analyzed using three- and two-way ANOVA using SAS version 9.4; significance was set at *P* < 0.05.

### Ethics approval and consent to participate

Plant sample collection was performed in accordance with the relevant guidelines and regulations such as “The IUCN Policy Statement on Research Involving Species at Risk of Extinction” and “The Convention on the Trade in Endangered Species of Wild Fauna and Flora”.

## Results

### Survival of thawed cultured gametophytes after cryopreservation

**Figure 1:**
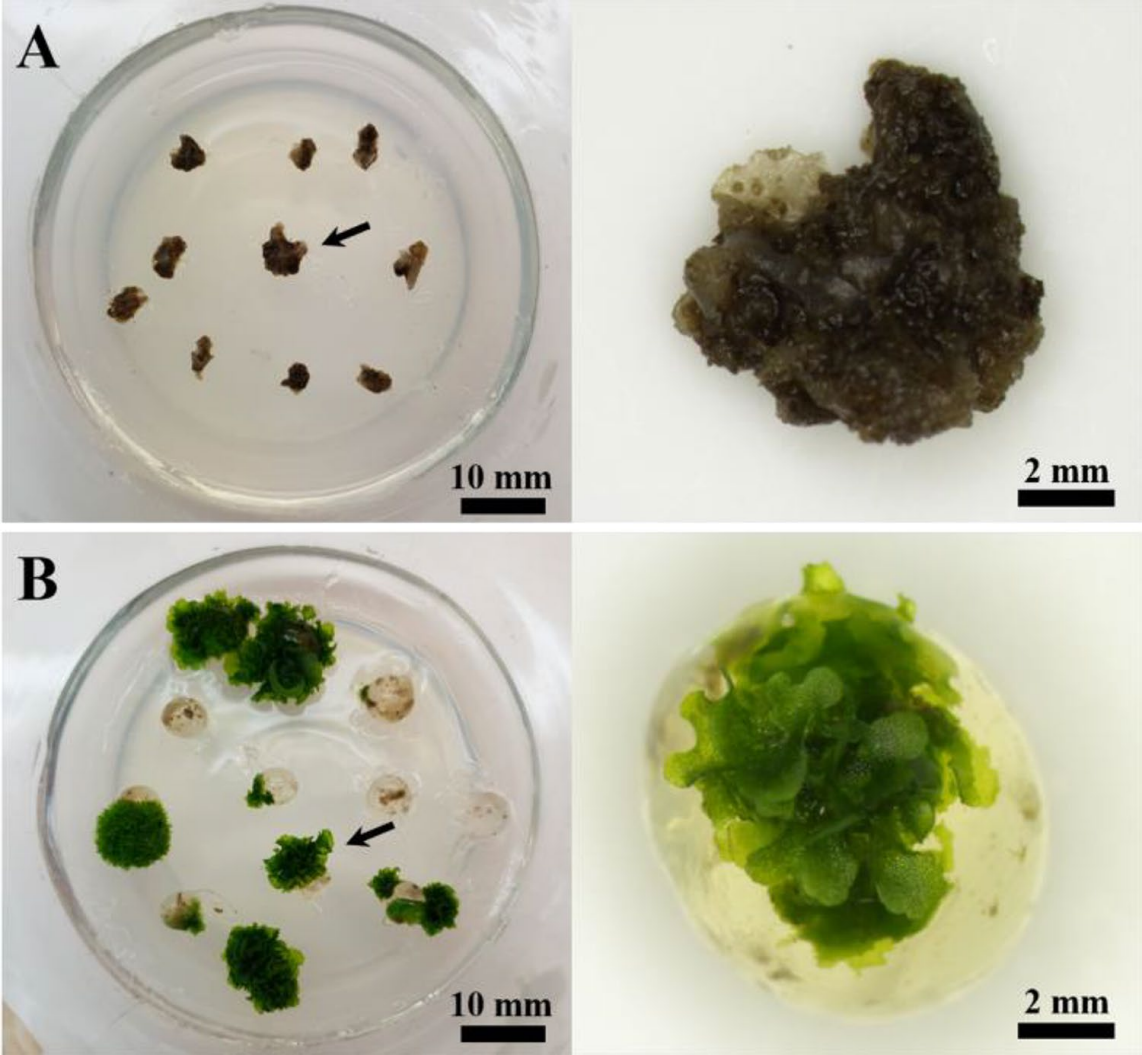
Recovery culture of encapsulation-dehydration-treated eastern bracken gametophytes after 4 weeks. (**A**) gametophytes without encapsulation; (**B**) gametophytes with encapsulation.

Unencapsulated gametophytes did not survive the exposure to LN regardless of the loading and drying time durations (Table 2). Alginate bead-encapsulated gametophytes showed a tendency to survive only after drying for ≥ 4 h regardless of the loading time. After 4 weeks of recovery culture, unencapsulated gametophyte cells died, whereas the encapsulated gametophytes regenerated normally (Fig. 1). Particularly, alginate bead-encapsulated gametophytes showed 74.0% survival after an 18 h loading and 6 h drying period. This was the highest survival rate observed in this study. These results show that encapsulation, loading time, and drying time significantly affected gametophyte survival after LN exposure and showed a highly significant correlation.

**Table 2:** Cryopreservation of eastern bracken fern gametophytes via the encapsulation–dehydration method at different loading and drying times.

Conservation methods	Loading time (h)	Survival ratio (%)			
		Drying time (h)			
		0	2	4	6
Untreated	0	0.0	0.0	0.0 d^z^	0.0 d
	12	0.0	0.0	0.0 d	0.0 d
	18	0.0	0.0	0.0 d	0.0 d
	24	0.0	0.0	0.0 d	0.0 d
Encapsulation	0	0.0	0.0	2.0 ± 2.00 d	2.0 ± 2.00 d
	12	0.0	0.0	0.0 d	12.0 ± 3.74 cd
	18	0.0	0.0	14.0 ± 9.80 c	74.0 ± 2.45 a
	24	0.0	0.0	14.0 ± 5.10 c	60.0 ± 17.03 b
Significance					
Method (A)	***				
Loading (B)	***				
Drying (C)	***				
A × B	***				
A × C	***				
B × C	***				
A × B × C	***				

^z^Different letters indicate a significant difference as per Duncan’s multiple range test at *P* < 0.05.

***significance at *P* < 0.001.

### Determination of water content of encapsulated gametophytes

**Figure 2:**
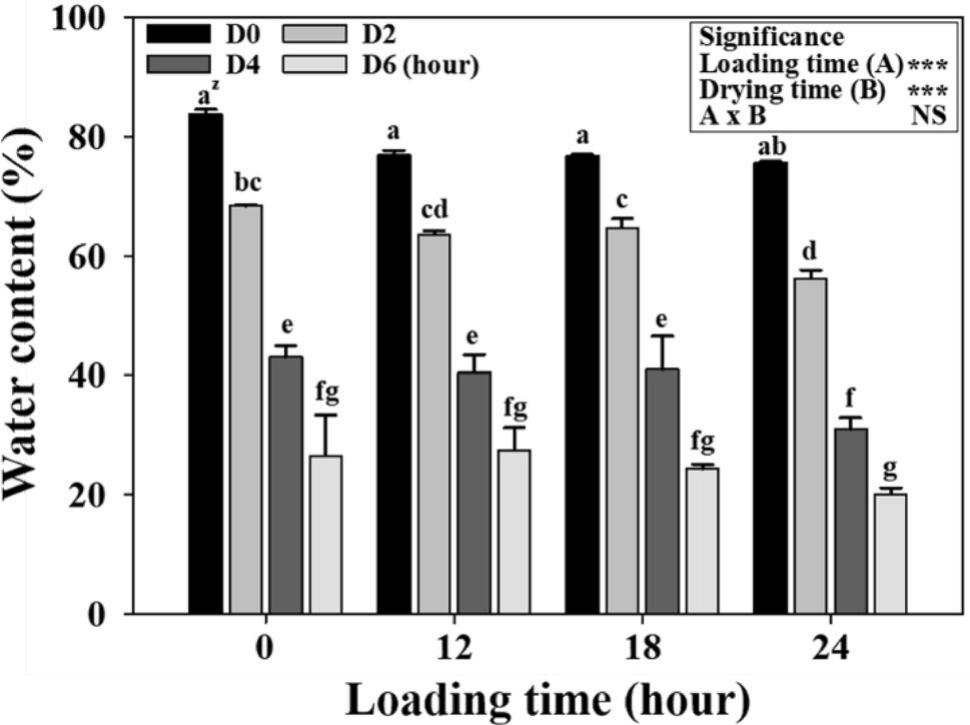
Changes in water content due to encapsulation-dehydration at varying loading and drying times in eastern bracken gametophytes. Vertical bars represent mean ± standard error (*n* = 3). ^z^Different letters indicate a significant difference as per Duncan’s multiple range test at *P* < 0.05. *NS* not significant, ***significance at *P* < 0.001.

**Figure 3:**
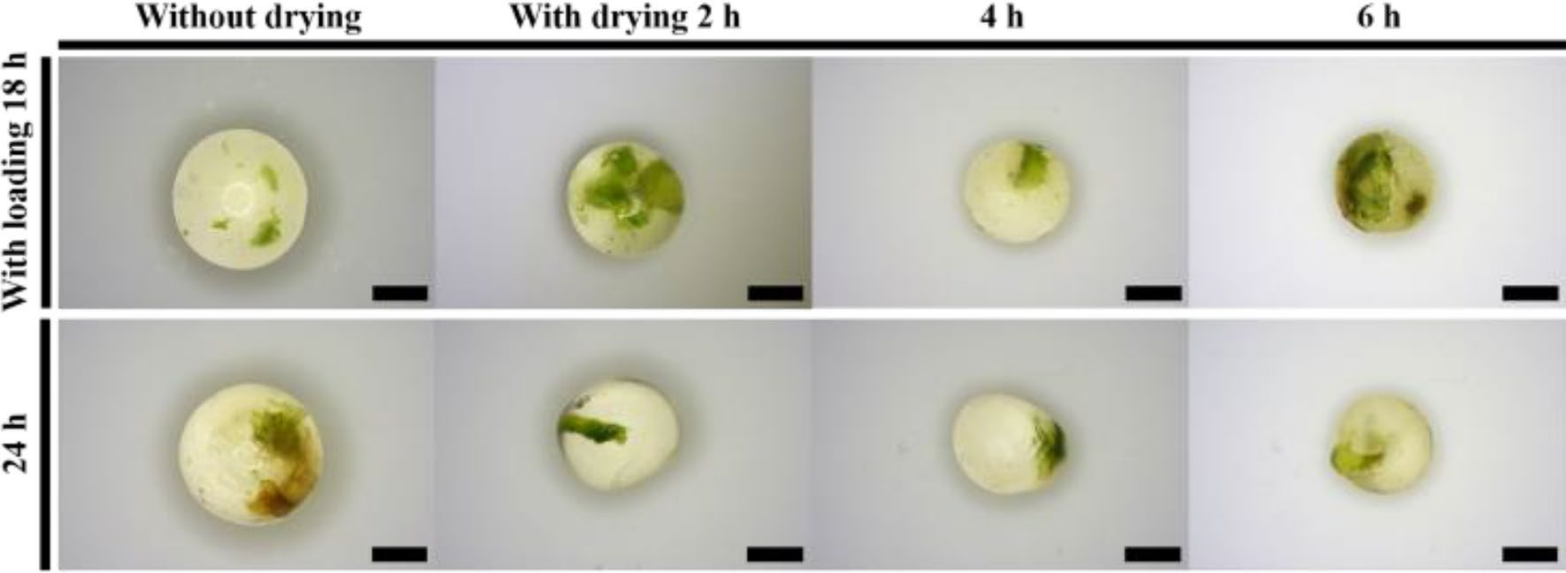
The shape of encapsulated gametophyte beads in the encapsulation-dehydration method depending on loading and drying times in eastern bracken gametophytes. Scale bars = 2 mm.

The total water content in the alginate beads (which includes that of the gametophytes) was investigated differently depending on loading and drying times (Fig. 2). Untreated alginate beads initially contained 83.7% water, which reduced to 8.0% after 24 h of loading treatment. Similarly, drying treatment significantly reduced the water content to 68.4, 43.0, and 26.6% after drying for 2, 4, and 6 h, respectively. Survival was confirmed based on the drying treatment, which differed depending on the loading time; however, the water content in the beads was determined at 4 h (31.0−43.0%) and 6 h (20.1−27.5%). Drying was also visually observed as wrinkling on the surface of the alginate beads and size reduction (Fig. 3).

### Enhanced survival and plant regeneration of gametophyte after preculture

**Figure 4:**
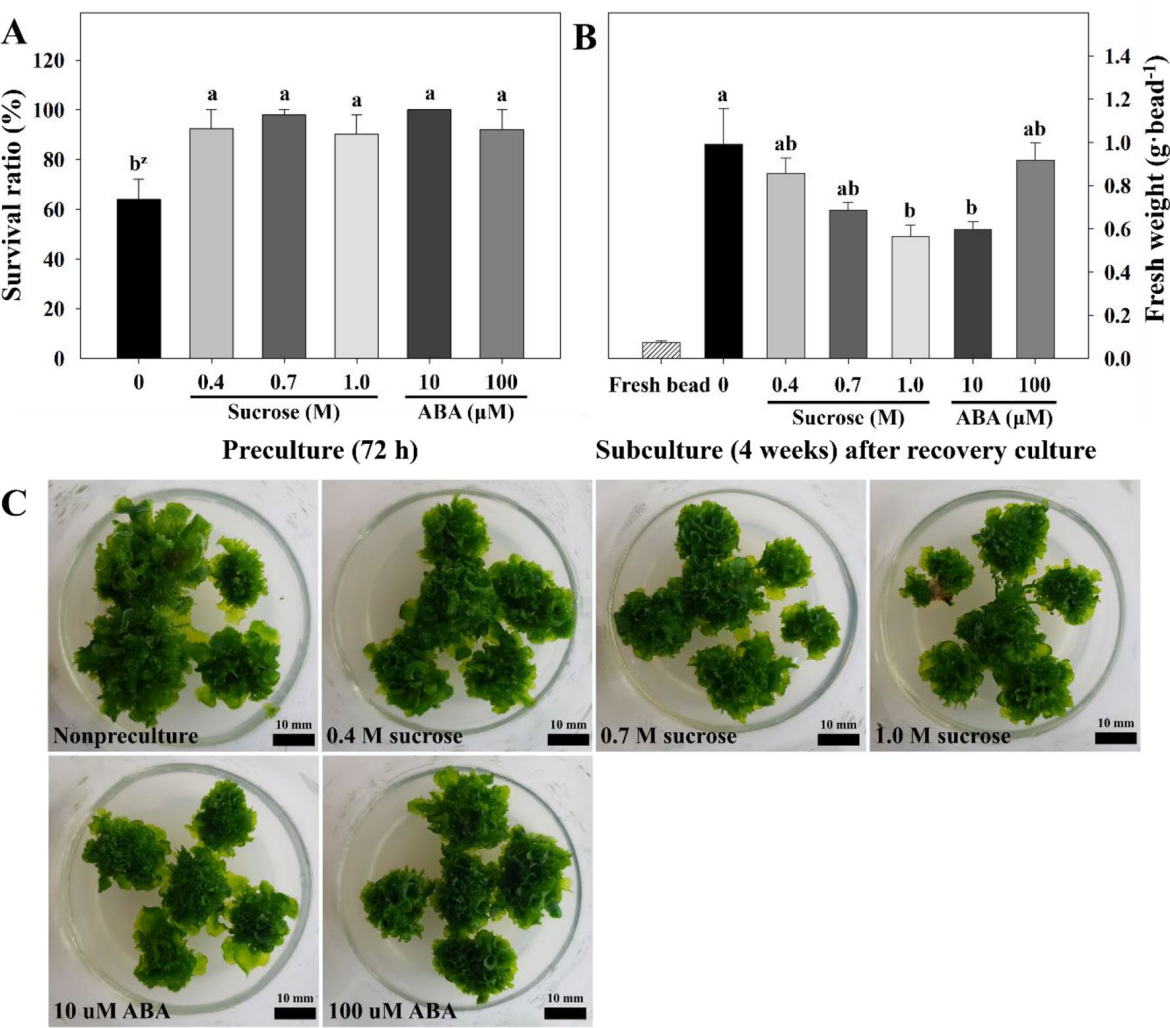
Cryopreservation of eastern bracken gametophytes enhanced by sucrose and ABA preculture. (**A**) survival ratio enhanced by preculture; (**B**) and (**C**) regenerated gametophytes encapsulated for 4 weeks after 4 weeks of recovery culture. Vertical bars represent mean ± standard error (a, *n* = 5; b, *n* = 10). ^z^Different letters indicate a significant difference as per Duncan’s multiple range test at *P* < 0.05.

**Figure 5:**
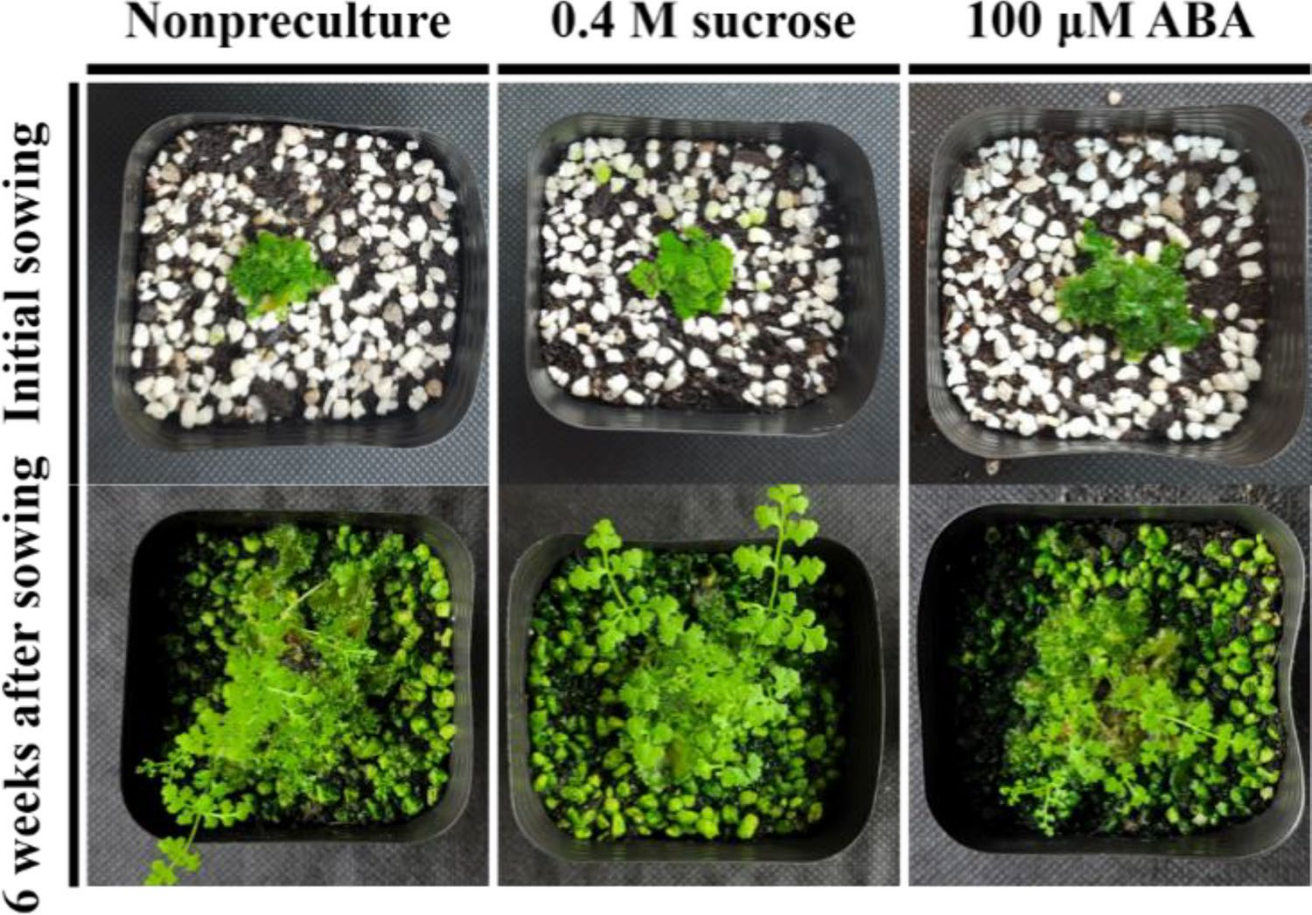
Sporophytes formed from regenerated eastern bracken gametophytes in ex vitro soil.

Preculture with sucrose and ABA significantly improved gametophyte survival after cryopreservation (Fig. 4A). The survival ratio of gametophytes increased to 90.2 − 100% with preculture, whereas the survival rate without preculture was 64 ± 8.1%. Survival rates did not significantly differ based on the sucrose and ABA concentrations used in preculture. Meanwhile, alginate bead-encapsulated gametophytes that were subcultured in 2MS medium following recovery culture proliferated normally after 4 weeks (Fig. 4B,C). The fresh weight of the cultured gametophytes increased significantly compared with the initial fresh weight in the alginate beads (74 ± 7 mg). Although treatment without preculture resulted in a relatively low survival ratio, it showed the highest fresh weight per alginate bead at 992 mg. Moreover, as sucrose concentration (0.4, 0.7, 1.0 M) increased, the fresh weight (857, 686, 565 mg) decreased. In contrast, an increase in ABA concentration (10, 100 μM) increased the fresh weight (598, 918 mg). Subsequently, the gametophytes subjected to regeneration were sown in soil *ex vitro*, and the gametophytes that regenerated successfully after cryopreservation formed sporophytes normally (Fig. 5).

### Gene expression of *PaSuSy*, *PaABI1b*, and *PaLEA14* in gametophytes after preculture and cryopreservation

**Figure 6:**
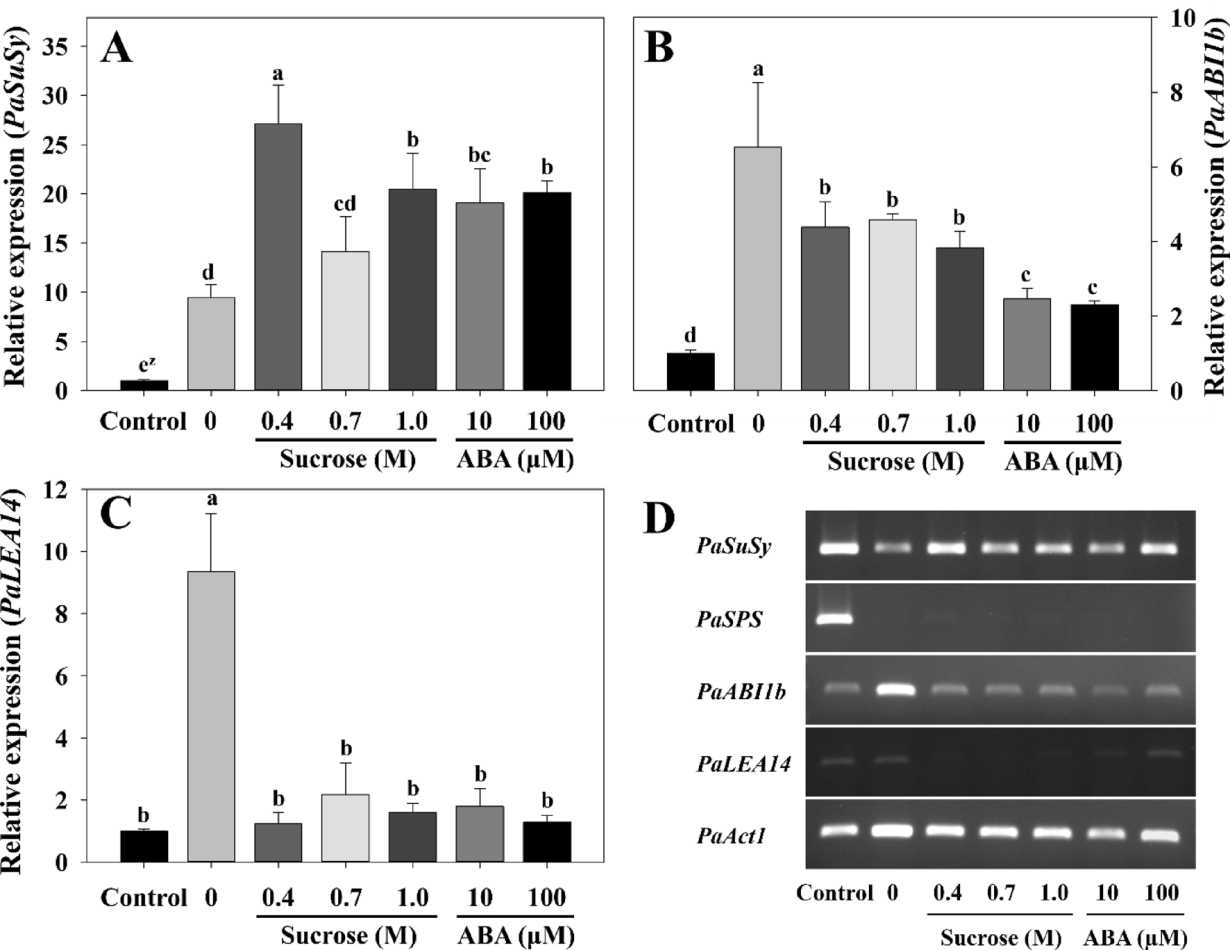
Relative expression of *PaSuSy* (**A**), *PaABI1b* (**B**), *PaLEA14* (**C**), and reverse transcription PCR results (cropped) of four target genes and *PaAct1* (**D**) in the cryopreserved eastern bracken gametophytes after preculture. Control, non-treated fresh gametophytes. Vertical bars represent mean ± standard deviation (*n* = 3). ^z^Different letters indicate a significant difference as per Duncan’s multiple range test at *P* < 0.05. The original agarose gel electrophoresis is presented in Supplementary Figs. 1–4.

*PaSuSy* and *PaABI1b* were upregulated in all treatment groups compared with those in the control (non-treated fresh gametophytes) (Fig. 6). *PaSuSy* gene expression in the sucrose and ABA preculture groups was relatively higher than that in the non-preculture treatment group. Preculture treatment with 0.4 M sucrose induced the highest gene expression (Fig. 6A). In contrast, *PaLEA14* and *PaABI1b* expression was downregulated in the sucrose and ABA preculture treatment groups compared with that of the non-preculture group (Fig. 6B,C). However, *PaLEA14* expression level did not differ between the two preculture treatments, although higher *PaABI1b* expression was observed in the sucrose treatment group than in the exogenous ABA treatment group with respect to preculture. The reverse transcription PCR results (Fig. 6D and Supplementary Figs. S1–S4) showed that *PaSPS* expression was not observed in any of the treatment groups.

### Endogenous ABA content after preculture and cryopreservation of gametophytes

**Figure 7:**
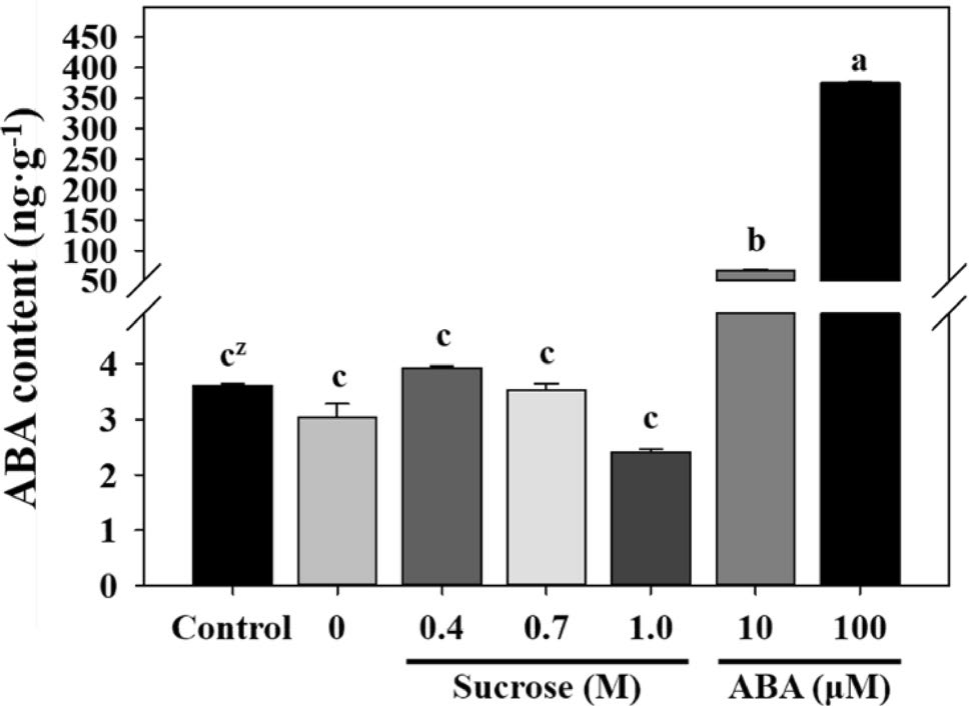
Endogenous ABA content of eastern bracken gametophytes enhanced by sucrose and ABA preculture. Control, fresh gametophytes that have not undergone encapsulation or cryopreservation. Vertical bars represent mean ± standard deviation (*n* = 3). ^z^Different letters indicate a significant difference as per Duncan’s multiple range test at *P* < 0.05.

Exogenous ABA preculture treatment induced high levels of endogenous ABA (Fig. 7). The ABA content in the control was 3.6 ng·g^−1^, whereas those of the 10 and 100 μM ABA preculture treatment samples were significantly higher at 66.9 and 375.0 ng·g^−1^, respectively. In contrast, the endogenous ABA contents of the non-preculture and sucrose preculture treatment samples were similar and ranged from 2.4–3.9 ng·g^−1^.

## Discussion

The encapsulation–dehydration method of preservation has been applied to various plant species, and gametophytes of six fern species have been successfully cryopreserved by Pence^19,47^. In the present study, the unencapsulated gametophytes did not survive and the water content in the encapsulated gametophytes differed depending on the duration of dehydration and drying, which considerably affected survival. Thus, dehydration, drying, and water content are key factors that must be carefully monitored to achieve a high survival rate following cryopreservation. In fact, their effects have been demonstrated in the preservation of various ferns, including *Osmunda regalis*^48^, *Lepisorus longifolius*, *Pteris adscensionis*^49^, *Cyathea dealbata*, *Dicksonia fibrosa*, *Phyllitis scolopendrium*^50^ and *Asplenium scolopendrium* var. *americanum*^51^. If the cells are not sufficiently dehydrated, they would be damaged from cryoinjury due to intracellular ice crystal formation, whereas excessive dehydration causes damage by desiccation and osmotic stress^52^. Hence, although dehydration and drying are essential to achieve the highest survival rate in encapsulated samples, data suggest that an appropriate range exists for the preferred water content during cryopreservation. As the moisture content in seeds and spores before cryopreservation affects survival rates after cryopreservation, it was adjusted to an appropriate level to minimize losses due to cryopreservation in plants such as *Pteris vittata* (0.025 g H_2_O/g dry weight), *Polystichum setiferum* (0.039 g H_2_O/g dry weight)^53^, *Prunus avium* (9.0–16.9%), *Prunus armeniacal* (7%), and *Paeonia emodi* (18.33%)^4,54,55^. Additionally, maintaining a 5.0–5.4% moisture content in eastern bracken spores before cryopreservation was found to be effective^7^. In the present study, a sample with a high survival rate of 74.0% was found to have a water content of ≤ 27.5%, proving that the encapsulation–dehydration method is suitable for the long-term conservation of eastern bracken gametophytes.

Generally, abiotic stress increases sucrose levels in plants^32^ while simultaneously regulating the expression of genes involved in sucrose synthesis and metabolism^30^. In the present study, sucrose preculture enhanced survival, suggesting a protective role for sucrose in shielding cells from damage caused by dehydration, desiccation, and freezing, particularly in relation to *PaSuSy* expression. In fact, strong abiotic stress (dehydration, drought, and freezing) during cryopreservation upregulated *PaSuSy* expression as did exogenous sucrose and ABA preculture. Upregulation of *SuSy* by abiotic stress has been reported in various plant species including *Arabidopsis thaliana*^56^, *Glycine max* L.^57^, *Gossypium hirsutum* L.^58^ and *Zea mays*^30^ Meanwhile, *PaSuSy*, which plays a role in sucrose synthesis and cleavage in the sucrose degradation pathway, may have been upregulated to a higher degree than *PaSPS* because the cells had a sufficient supply of sucrose. Thus sucrose is a key factor for the successful cryopreservation and revival of eastern bracken gametophytes.

Various treatments in the cryopreservation process (osmosis, dehydration, ice crystal formation during freezing, and thawing) can act as strong plant cell stressors^59^. The stress of cryopreservation significantly increased the expression of *PaLEA14* in the non-preculture samples than in the control. The activation of LEA or LEA-like proteins is upregulated by various abiotic stresses^60^ and it improves dehydration- and desiccation tolerance^61,62^. The proteins encoded by *LEA* and *LEA-*like gene groups, including *LEA1*, *LEA2*, *LEA3*, *LEA4*, *LEA5*, *LEA6*, *LEA7*, and *LEA8*, may exhibit different expression and gene regulation patterns^63^. ABA is involved in LEA-like protein accumulation and improves stress tolerance in the liverwort *Marchantia polymorpha*^26^. Exogenous ABA upregulated 14 *LEA-*like transcripts in Linderniaceae species^64^ and *LEA* expression in *Solanum tuberosum*^61^. However, in the present study, exogenous sucrose and ABA preculture decreased *PaLEA14* expression. This trend of decreased expression level is contradictory to the high survival rate observed in the samples, suggesting that preculture offsets a considerable amount of stress exerted by the cryopreservation process. Stress alleviation through preculture has been reported in various plant species. Preculture with exogenous ABA had a positive effect on osmotic stress and the activities of caspase-3-like and caspase-9-like enzymes that characterize programmed cell death. This resulted in improved survival of *Lilium pumilum* shoot tips after cryopreservation^65^. Additionally, Htwe et al.^59^ analyzed transcriptome data obtained from banana shoot meristem subjected to high sucrose pretreatment before cryopreservation and reported differential expression in genes related to osmotic and oxidative stress.

Similar to the effect on *PaLEA14*, stress due to the cryopreservation process significantly increased *PaABI1b* expression in non-preculture samples. *ABI1* encodes protein serine/threonine phosphatases (PP2C), which is a monomeric enzyme that is involved in ABA signaling^66^. The PP2C group A in *Arabidopsis*, including ABI1, ABI2, and AtPP2CA, has been studied extensively and is recognized as a negative regulator of ABA signaling and responses^66,67^. Komatsu et al.^68^ reported two PP2C groups similar to *ABI1* in *Physcomitrella patens*, and the freezing tolerance of the *Arabidopsis abi1-1* mutant decreased significantly at higher expression levels of this gene. In the present study, the expression level of *PaABI1b* was higher in non-preculture samples than in the sucrose and ABA preculture samples. This result implies that preculture enhances dehydration- and freezing tolerance and survival and that the *ABI1* gene acts as a negative regulator of ABA signaling in the eastern bracken gametophyte. Furthermore, *PaABI1b* exhibited a relatively low expression level in exogenous ABA preculture, which was opposite to the effect observed for endogenous ABA content. Verslues and Bary^69^ reported that *ABI1* elevates ABA content in response to exogenous ABA. Furthermore, Lu et al.^70^ reported that *ABI1* serves as a crucial negative regulator of ABA signaling, impeding ABI1 function in the presence of ABA. These results indicate that negative regulators of ABA signaling induce ABA accumulation and may explain the role of negative regulators of ABA signaling.

## Conclusions

In conclusion, the results of this study indicate that long-term conservation can be achieved in eastern bracken gametophytes through cryopreservation. On one hand, the encapsulation–dehydration method acts as a strong abiotic stress on the gametophyte cells. On the other hand, it shields the cells against dehydration, desiccation, and freezing (Fig. 6). Exogenous sucrose and ABA preculture increased survival and regulated the expression of genes related to stress and endogenous ABA content. The expression of *PaSuSy*, which is involved in sucrose synthesis and metabolism, was upregulated. The considerable stress due to the cryopreservation process significantly increased *PaLEA14* and *PaABI1b* expression levels in non-preculture samples. However, *PaABI1b* was relatively downregulated in exogenous ABA preculture and showed an opposite trend to that of endogenous ABA accumulation. These results indicate that the *PaABI1b* gene augments the role of the negative regulators of ABA signaling in eastern bracken gametophytes. The results of this study can potentially contribute to better cryopreservation protocols for additional fern species.

### Supplementary Information

Supplementary Figure S1.

Supplementary Figure S2.

Supplementary Figure S3.

Supplementary Figure S4.

## Data Availability

The datasets used and/or analyzed during the current study are available from the corresponding author on reasonable request.
