# Supplementary material for: Exogenous preculture with sucrose and abscisic acid improves post-cryopreservation survival of eastern bracken fern gametophytes
Source: Sci Rep. 2023 Oct 28;13:18518. doi: 10.1038/s41598-023-45941-3 (PMC10613233; doi:10.1038/s41598-023-45941-3)
Supplement: Supplementary file 2 — Supplementary Figure S2. [file 41598_2023_45941_MOESM2_ESM.docx]

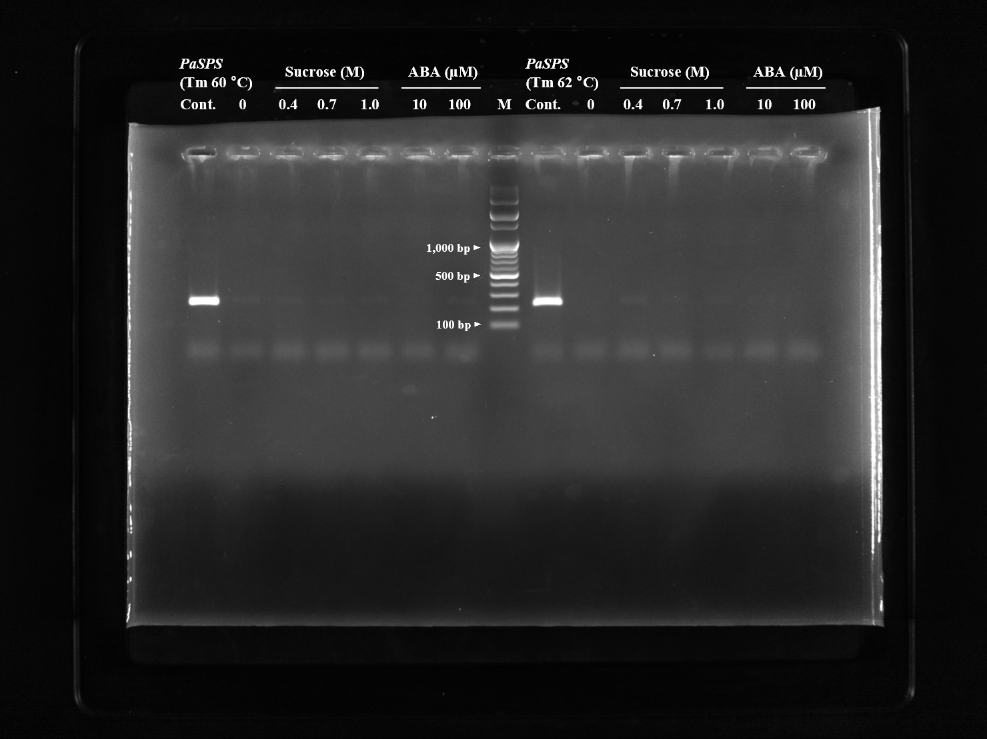


**Figure S2**. Reverse transcription PCR results of *PaSPS* in the cryopreserved eastern bracken gametophytes after preculture. Control (Cont.), non-treated fresh gametophytes; M, 100 bp ladder marker. The gel image data represent the result of reverse transcription PCR to confirm the production of amplicons with expected size and to compare the expression level with real-time qPCR. The *PaSPS* was analyzed twice with different annealing temperatures to determine the appropriate annealing temperature.
